# Rubidium Ions Enhanced Crystallinity for Ruddlesden–Popper Perovskites

**DOI:** 10.1002/advs.202002445

**Published:** 2020-11-13

**Authors:** Shaowen Cui, Jifei Wang, Haipeng Xie, Yuan Zhao, Zhimin Li, Shiqiang Luo, Lili Ke, Yongli Gao, Ke Meng, Liming Ding, Yongbo Yuan

**Affiliations:** ^1^ Hunan Key Laboratory of Super Microstructure and Ultrafast Process School of Physics and Electronics Central South University Changsha Hunan 410083 P. R. China; ^2^ School of Physical Science and Technology Shanghai Tech University Shanghai 201210 P. R. China; ^3^ Center for Excellence in Nanoscience (CAS) Key Laboratory of Nanosystem and Hierarchical Fabrication (CAS) National Center for Nanoscience and Technology Beijing 100190 P. R. China; ^4^ State Key Laboratory of Powder Metallurgy Central South University Changsha Hunan 410083 P. R. China

**Keywords:** additive, crystal growth rate, crystallinity, perovskite solar cells, Ruddlesden–Popper perovskites

## Abstract

Tailoring the organic spacing cations enables developing new Ruddlesden–Popper (RP) perovskites with tunable optoelectronic properties and superior stabilities. However, the formation of highly crystallized RP perovskites can be hindered when the structure of organic cations become complex. Strategies to regulate crystal growing process and grains quality remain to be explored. In this study, mixing Rb^+^ ions in precursor solution is reported to significantly promote the crystallinity of phenylethylammonium (PEA^+^) based RP perovskites without impacting on the major orientation of perovskite grains, which leads to increased power conversion efficiencies from 12.5% to 14.6%. It is found that the added Rb^+^ ions prefer to accumulate at crystal growing front and form Rb^+^ ions‐rich region, which functions as mild crystal growth inhibitor to retard the absorption and diffusion of organic cations at growing front and hence regulates crystal growing rate. The retarded crystal growth benefits PEA‐based RP perovskite films with elevated crystal qualities and prolonged carrier recombination lifetimes. Similar increased crystallinity and photovoltaic performance are achieved in other RP perovskites with non‐linear organic cations such as phenylmethylammonium (PMA^+^), 1‐(2‐naphthyl)‐methanammoniun (NMA^+^) by adding Rb^+^ ions, demonstrating using a small amount of growth inhibitor as a general route to regulate crystal growth.

## Graphical abstract

Besides achieving large grains by reducing the nucleation centers, controlling the crystal growth rate is another key issue to pursue high crystallinity. The crystal quality of quasi‐2D perovskite can be substantially improved by adding a small amount of growth inhibitors (i.e., Rb^+^ ions) in the precursor solution, which dynamically accumulate in the crystal growth front and retard the crystal growth rate.

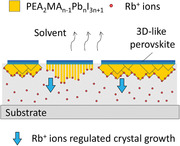

Layered perovskites such as Ruddlesden–Popper (RP) perovskites have been intensively studied in the past few years due to its better environmental stability^[^ ^1^, ^2^, ^3^, ^4^, ^5^, ^6^, ^7^ ^]^ and less trend of ion migration.^[^ ^8^, ^9^, ^10^ ^]^ Compared with its 3D counterparts, RP perovskite solar cells (PSCs) generally show lower power conversion efficiencies (PCEs) because of its worse conductivity and larger bandgap. Tailoring the organic cation with functional segments is considered as an important approach for developing layered perovskites with higher intrinsic charge transport properties or/and adjustable electronic structures.^[^ ^4^, ^5^, ^11^, ^12^, ^13^ ^]^ However, on the other hand, using organic cations with increased complexity would impact on the crystallization process of layered perovskites due to steric effect or/and intermolecular interactions.^[^ ^11^, ^12^ ^]^ Currently, among a lot of synthesized bulky cations, the most widely used organic cations in RP perovskites are phenylethylammonium (PEA^+^) and butylammonium (BA^+^).^[^ ^2^, ^4^, ^5^, ^14^, ^15^, ^16^ ^]^ Recently, the highest PCE value (18.04%) for PEA‐based RP PSCs was obtained by forming an energy‐cascading structure through vacuum poling.^[^ ^17^ ^]^ Meanwhile, it still can be noticed that the reported PCE values of most of the PEA‐based RP PSCs (≈13%^[^ ^18^, ^19^, ^20^, ^21^, ^22^, ^23^ ^]^) are generally lower than that of BA‐based RP PSCs (>14%^[^ ^24^, ^25^ ^]^) in the past years although it was the first one being applied into the field of solar cells.^[^ ^4^ ^]^ Besides, the PCEs of RP PSCs based on other non‐linear organic cations beyond PEA^+^, such as PMA^+^, NMA^+^ and etc., are also need to be improved.

In general, issues like crystal orientation and crystallinity largely determine the PCEs of RP perovskite solar cells. Realizing out‐of‐plane orientation of RP perovskite crystals now is widely considered as a prerequisite for high efficiency solar cells as it addresses the carrier transport problems. Up to now, methods to promote out‐of‐plane dominated orientation of RP perovskite crystals include: hot casting,^[^ ^26^, ^27^, ^28^ ^]^ additive assistant,^[^ ^24^ ^]^ and solvent engineering.^[^ ^29^, ^30^ ^]^ Recent advances revealed that the out‐of‐plane dominated orientation resulted from the templated growth of RP perovskites from preformed 3D‐like is available to varied bulky organic cations with different shapes. While knowledge for controlling the orientation of RP perovskite crystals has been largely enriched, investigations on manipulating the quality of RP perovskite crystals and its impacts on the PCEs of PSCs are rarely reported.^[^ ^31^ ^]^ Generally, grains with out‐of‐plane dominated orientation do not necessarily exhibit high crystallinity. This is because regions with structural disorders, which are embedded in grains without producing additional X‐ray diffraction (XRD) peaks, are still possible.^[^ ^32^ ^]^ In this study, we report adding a small amount of crystal growth inhibitor in solution as an effective and reproducible approach to enhance the PCEs of PEA‐based RP perovskite solar cells from 12.5% to 14.6%. The obtained (PEA)_2_(MA)_4_Pb_5_I_16_ perovskite films with Rb^+^ ions as crystal growth inhibitor exhibited much improved crystallinity and prolonged carrier recombination lifetime, explaining the enhanced efficiencies. The underlying mechanism for the Rb^+^ ions regulated crystallinity has been studied in details.

In this work, PEA‐based RP perovskite (PEA)_2_(MA)*_n_*_−1_Pb*_n_*I_3_ *_n_*_+1_ (*n* = 3, 4, and 5) films are prepared by a one‐step spin coating method with NH_4_Cl as additive.^[^ ^33^ ^]^ Similar to the mechanism reported in our previous study, the role of NH_4_Cl was to promote the downward growth of PEA‐based RP perovskite, as illustrated in **Figure** **1**a. We introduced a small amount of RbI into the perovskite precursor solution and fabricated the PEA‐based RP perovskite films to investigate its influence on the crystallization process. The optical absorption spectra of the films without or with Rb^+^ ions were compared in Figure 1b. When 5% RbI (molar ratio of RbI to PbI_2_) was added into the precursor solution, the resulted films showed stronger excitonic absorption peaks at wavelength of 565, 606, and 642 nm, which proves the formation of layered perovskite phases with *n* = 2, 3, and 4, respectively. These increased excitonic absorption peaks suggest a better crystallinity of the layered perovskites after adding Rb^+^ ions.

**Figure 1:**
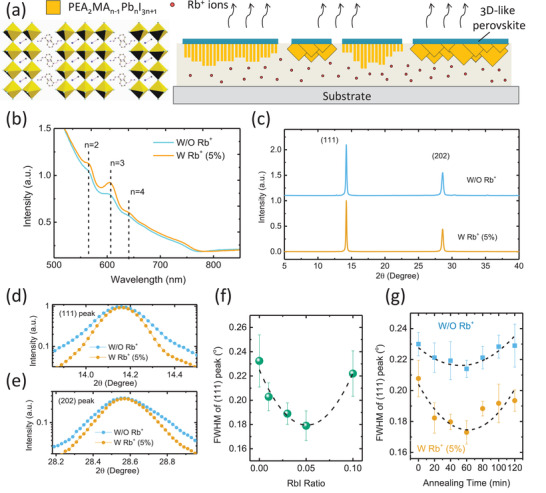
a) Illustration of downward growth of PEA‐based RP perovskite induced by using NH_4_Cl as additive. b) UV–vis absorption spectra of (PEA)_2_(MA)_4_Pb_5_I_16_perovskite films without and with 5% Rb^+^ions (molar ratio) added in the precursor solution. c) X‐ray diffraction (XRD) spectra of the (PEA)_2_(MA)_4_Pb_5_I_16_films without and with 5% Rb^+^ions. d,e) Comparison of the (111) and (202) diffraction peaks of PEA‐based RP perovskites without and with 5% Rb^+^ions in the precursor solution. f) The FWHM of the (111) diffraction peaks of (PEA)_2_(MA)_4_Pb_5_I_16_perovskite films with different amounts of Rb^+^ions added in the precursor solution. g) The FWHM of the (111) diffraction peaks of (PEA)_2_(MA)_4_Pb_5_I_16_perovskite films with different thermal annealing time (at 100 °C).

To further study the impact of Rb^+^ ions on the crystal orientation and crystallinity of RP perovskite films, X‐ray diffraction (XRD) characterizations of the (PEA)_2_(MA)_4_Pb_5_I_16_ (<*n*> = 5) films with different amounts of RbI had been carried out (Figure 1c and Figure S1, Supporting Information). All samples showed two dominant diffraction peaks at 2*θ* = 14.2° and 28.5°, which can be assigned to the (111) and (202) planes of PEA‐based RP perovskites. Meanwhile, the absence of peaks at diffraction angle below 14° suggests most of the (PEA)_2_(MA)_4_Pb_5_I_16_ adopt out‐of‐plane orientation, which holds true for both RP perovskite without and with Rb^+^ ions. The out‐of‐plane orientation can be further verified for all RP perovskite samples by the grazing incidence wide angle X‐ray scattering (GIWAXS) patterns (Figure S2, Supporting Information), indicating that adding Rb^+^ ions (from 0% to 10%) has no impact on the grain orientation. There were no peak shift at the both (111) and (202) diffraction peaks of the perovskite films after adding Rb^+^ ions (Figure 1d,e), suggesting that no lattice contraction took place and these Rb^+^ ions did not occupy the A‐site of the PEA‐based RP perovskite lattice, which is different from the case of cesium cation (Cs^+^) doping in BA‐based RP perovskite as reported previously.^[^ ^26^ ^]^ This is because the incorporation of Rb^+^ with significantly smaller ion radius into corner‐sharing PbI_6_ octahedra networks is thermodynamically unfavorable due to the distortion from the Goldschmidt tolerance factor.^[^ ^34^ ^]^ Nevertheless, after adding Rb^+^ ions, the crystallinity of the (PEA)_2_(MA)_4_Pb_5_I_16_ films has been improved, as indicated by the reduced full‐width at half maximum (FWHM) of the (111) and (202) diffraction peaks shown in Figure 1d,e, respectively. The FWHM of the (111) diffraction peak reduced gradually from 0.24° to 0.18° when the Rb^+^ ions increased from 0% to 5% (Figure 1f), but further increasing the Rb^+^ ions from 5% to 10% made the FWHM value increased from 0.18° to 0.22°. The over three‐times enhanced intensity of the (111) diffraction peak in the GIWAXS patterns also confirmed the much enhanced crystallinity of RP perovskite after adding Rb^+^ ions (Figure S2f, Supporting Information). No clear morphology changes in these (PEA)_2_(MA)*_n_*_−1_Pb*_n_*I_3_ *_n_*_+1_ films (<*n*> = 3, 4, and 5) can be resolved after adding Rb^+^ ions, as identified from the cross‐sectional scanning electron microscopy (SEM) images (Figure S3, Supporting Information), which suggests that the crystallinity improvement introduced by 5% Rb^+^ ions mainly originates from increased crystal quality rather than grain size. The similar average grain sizes also suggested that the added 5% Rb^+^ ions do not influence the density of nucleation centers significantly, as the nucleation is largely suppressed by the NH_4_Cl additives.^[^ ^25^ ^]^

Since thermal annealing has great influence on the film crystallinity, we further investigated a series of control samples and Rb^+^‐contained samples with different thermal annealing times to exclude the FWHM variation caused by thermal annealing. Figure 1g clearly shows that the FWHM of (PEA)_2_(MA)_4_Pb_5_I_16_ with Rb^+^ ions is much narrower than that of the pristine ones in the entire annealing time range of 0–120 min. Optimized thermal annealing can reduce the FWHM of samples without Rb^+^ ions from 0.23° to a minimum value of 0.21°. By contrast, adding Rb^+^ ions can further reduce the FWHM value to 0.17°. These results prove that adding Rb^+^ ions in precursor solution can significantly promote the crystallinity of PEA‐based RP perovskite films, which is irreplaceable by sole thermal annealing.

To explore the effect of added Rb^+^ ions on the photovoltaic performance of PSCs, devices with p‐i‐n planar structure of ITO / PEDOT: PSS / PEA_2_(MA)*_n_*_−1_Pb*_n_*I_3_ *_n_*_+1_ (*n* = 3, 4, and 5) / PC_61_BM / BCP / Cu were fabricated (**Figure** **2**a). For PEA‐based RP perovskites with an average layer number of <*n*> = 5, a significant improvement on *V*_OC_ from 1.13 to 1.22 V was observed when 5% Rb^+^ ions were added (Figure 2b). The *J*_SC_ of these two kinds of PSCs are nearly the same (Table S1, Supporting Information), which are consistent with the photocurrent values integrated by the external quantum efficiency (EQE) spectra in Figure 2c. The photocurrents are mainly contributed by the wavelength range of 350–650 nm, further proving that the obtained perovskite films are dominated by RP perovskite phases with small *n*‐value rather than large *n*‐value or 3D perovskite. Both kinds of devices show ignorable hysteresis in photocurrent, suggesting ignorable trends of ion migration.^[^ ^9^, ^13^ ^]^ The champion PCEs of PEA‐based PSCs (<*n*> = 5) enhance from original 12.5% to 14.6% after adding Rb^+^ ions (Figure S4, Supporting Information). In previous study, Cs^+^ doped (BA)_2_(MA)_3_Pb_4_I_13_ perovskite solar cells gave a power conversion efficiency (PCE) as high as 13.7%.^[^ ^26^ ^]^ As a major difference, the Cs^+^ ions were incorporated into the lattice of BA‐based RP perovskite and the PCE improvement mainly resulted from increased grain size by Cs^+^ ions doping which offered an increased *J*_SC_ in that work.^[^ ^26^ ^]^

**Figure 2:**
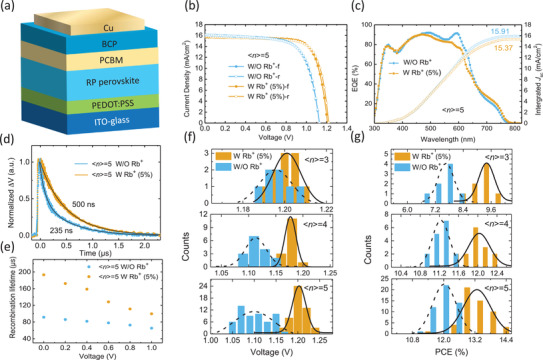
a) Scheme of the structure of RP PSC. b) Comparison of current density–voltage (*J–V*) curves of (PEA)_2_(MA)_4_Pb_5_I_16_without and with 5% Rb^+^ions under 100 mW cm^−2^AM1.5G illumination. c) EQE spectra of the PEA‐based PSCs (<*n*> = 5) without and with 5% Rb^+^ions. d) Transient photovoltage of PEA‐based PSCs (<*n*> = 5) without and with 5% Rb^+^ions. e) Plots of carrier recombination lifetime (obtained by EIS fitting) against applied bias of PEA‐based PSCs (<*n*> = 5) without and with 5% Rb^+^ions. f, g) Statistical distribution of the f)*V*_OC_and g) PCE values of PEA‐based PSCs (<*n*> = 3, 4, and 5) without and with 5% Rb^+^ions.

In order to obtain more insights into the origin of the improved *V*_OC_, the transient photovoltage (TPV) measurement was carried out (Figure 2d) on PEA‐based PSCs with <*n*> = 5 under open‐circuit condition and AM1.5G illumination. After adding Rb^+^ ions, the TPV lifetime of solar cells was increased from 235 to 500 ns, suggesting the recombination centers induced by defects or structural disorders in the RP perovskite films are reduced after adding Rb^+^ ions, which can result in larger quasi‐Fermi level splitting and hence improved *V*_OC_. We used impedance modelling to further assess the charge recombination lifetimes at forward bias, the electrochemical impedance spectroscopy (EIS) characterizations of these devices were carried out at different working biases of from 0 to 1.0 V and corresponding Nyquist plots (Figure S5, Supporting Information) were analyzed.^[^ ^29^, ^30^ ^]^ Compared to control devices, solar cells with 5% Rb^+^ ions show significantly longer recombination lifetime over the entire measured bias range (Figure 2e). This result effectively supports the conclusion that the reduced recombination centers in RP perovskite films with Rb^+^ ions is responsible for the higher PCE, in good agreement with the increased crystallinity shown in Figure 1d–g.

To further verify the PCE enhancement effect caused by added Rb^+^ ions, we systematically studied PEA‐based PSCs with average layer number of <*n*> = 3–5 (Figure 2f,g and Table S1, Supporting Information). In PSCs without Rb^+^ ions, the average value of *V*_OC_ distribution decreased gradually from 1.19 to 1.10 V with <*n*> increased from 3 to 5. By contrast, the average value of V_OC_ distribution kept at a level of ≈1.20 V by varying <*n*> from 3 to 5 in RP perovskite PSCs with 5% Rb^+^ ions. Accordingly, Figure 2g show that adding 5% Rb^+^ ions can result in solid PCE improvement in RP PSCs.

Since Rb^+^ ions do not remain in the bulk of RP perovskite grains due to its small ionic radius, it's quite necessary to find out the location of Rb^+^ ion in the resulted RP perovskite film. In our study, no X‐ray energy dispersive spectroscopy (EDS) signal of Rb elements can be detected from the top of spin‐coated RP perovskite films (**Figure** **3**a and Figure S6 and Table S2, Supporting Information). In striking contrast, we were able to find the EDS signal of Rb element from the bottom of the RP perovskite films with Rb/Pb ratio exceeding 5% (Figure 3a) by peeling the RP perovskite films off from the substrate, which qualitatively proved that Rb^+^ ions prefer to accumulate at the bottom side. Considering the EDS signal is averaged over a region of perovskite films as thick as tens of nanometers,^[^ ^35^, ^36^ ^]^ we further carried out X‐ray photoelectron (XPS) characterizations on each side of the film with a depth resolution of within 5 nm. While no XPS peak of Rb elements can be found on the top side, Rb 3d peak can be clearly observed at the bottom side of the RP perovskite film, which indicated a high Rb/Pb molar ratio of 30–40% on the surface of the bottom by integrating the area of Rb 3d and Pb 4f peaks (Figure 3b and Figure S7, Supporting Information), respectively. Moreover, after Ar^+^ ions (1500 eV) etching was introduced to bombard 10 nm thick perovskite layer near the bottom surface, the Rb 3d peak disappeared in situ. This study clearly proved that most of the Rb^+^ ions distributed at the bottom of the resulted RP perovskite film with a narrow region of a few nanometers.

**Figure 3:**
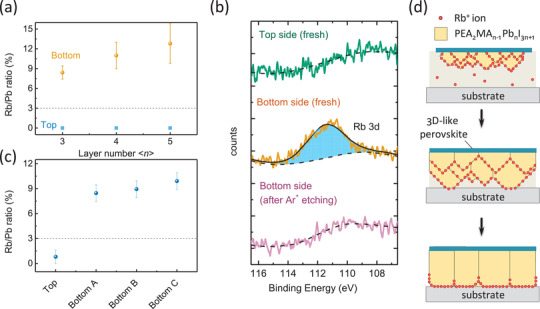
a) Comparison of Rb/Pb molar ratios of resulted RP perovskite films (<*n*> = 3, 4, and 5) by EDS characterization carried out from both top and bottom sides. b) High‐resolution XPS spectra of PEA‐based RP perovskite films (<*n*> = 5) with 5% Rb^+^ions measured at different conditions, where the Rb 3d peak at 111.2 eV indicates the presence of Rb element. c) Rb/Pb ratios on top or bottom of PEA‐based RP perovskite films at different time points (5, 10, and 30 min), during which the thickness of PEA‐based RP perovskite films increased continuously; d) Illustration of the dynamic distribution of Rb^+^ions during the crystallization process of PEA‐based RP perovskite along with solution thinning, during which Rb^+^ions prefer to accumulate at the crystal growth front.

To find out the dynamic evolution of Rb^+^ ions position during the film formation, we performed a top‐crust peeling‐off test. Recently we demonstrated that layered perovskite crystals nucleate under 3D‐like perovskite template that pre‐formed at the liquid‐air interface and grow in a downward mode (see Figure 1a),^[^ ^25^ ^]^ so that the crystal growth front actually propagate from top to bottom during solution thinning. Here we prove that Rb^+^ ions accumulate on the bottom of perovskite crystal (i.e., growth front) and propagate downward accordingly with the growth of PEA‐based RP perovskite grains. Similar to a top‐crust peeling‐off test reported previously,^[^ ^32^ ^]^ we dropped the precursor solution of RP perovskite with Rb^+^ ions on a warm substrate and let it dry slowly (Figure S8a, Supporting Information), during which the thickness of the RP perovskite film formed on top of the solution increased with time. At different time points of 5, 10, and 30 min, the RP perovskite films were peeled off and characterized by EDS measurement (Figure S8b–e and Table S3, Supporting Information). It turned out that during the downward growth of the RP perovskite, the EDS signal of Rb elements is ignorable on the film top but is always significant at the bottom of the RP perovskite films (Figure 3c). The averaged Rb/Pb ratios obtained at the bottom of RP perovskite films at different peeling times are kept at ≈9%, much higher than that in solution (3%), supporting our explanation that these Rb^+^ ions repelled from bulk of crystal tend to accumulate in growth front. Moreover, the XPS results show that these Rb^+^ ions at the bottom of RP perovskites are most likely to bond with PbI_6_ octahedra rather than forming pristine RbI salt, as indicated by the chemical shifts of Rb^+^ ions 3d peak from 110.4 eV (in pristine RbI phase) to 111.2 eV and I^−^ ions 3d_5/2_ peak from 619.5 eV (in RbI phase) to 620.1 eV (Figure S7, Supporting Information), i.e., these Rb^+^ ions preferred to bind with PbI_6_ octahedra on perovskite during solution thinning rather than purely dissolved in DMF and precipitated as last. Accordingly, the dynamic propagation of crystal growth front and Rb^+^ ions during film formation is illuminated in Figure 3d. The exact formula and crystal structure of the Rb^+^ ions‐rich region is not fully understood due to its very small thickness, which might contain inorganic RbPbI_3_ perovskite as reported by Jung et al.^[^ ^37^ ^]^

We found these Rb^+^ ions distributed in the crystal growth front are not inert but can regulate the crystal growth rate. In order to monitor the formation speed of perovskite crystals, an in situ transmittance characterization of the perovskite film (at a wavelength of 520 nm) was carried out, as illustrated in the inset of **Figure** **4**a (see details in Note S1, Supporting Information). The gradually decreased transparency starting at about 5th second shown in Figure 4a is attributed to the growing of perovskite grains during the solution thinning process. It turns out that adding Rb^+^ in precursor solution can slow down the crystallization of RP perovskite, and a higher Rb^+^ ions concentration can cause slower crystallization behaviour (Figure 4a). The photographs of RP perovskite films during spin coating process were also recorded (Figure S9, Supporting Information), which support that 5% Rb^+^ ions retard the formation of RP perovskite phase. In order to further visualize the retarded crystal growth, we soaked PbI_2_‐DMF solvated phase into the supersaturated precursor solution of PEA‐based RP perovskite, which would trigger directional templated growth of RP perovskite crystal on its surface as we reported previously.^[^ ^25^ ^]^ It was found that when Rb^+^ ions were added into the precursor solution, the size of RP perovskite flakes is apparently smaller than that of control sample (Figure 4b), confirming the crystal growth rate of the PEA‐based RP perovskite is retarded by Rb^+^ ions.

**Figure 4:**
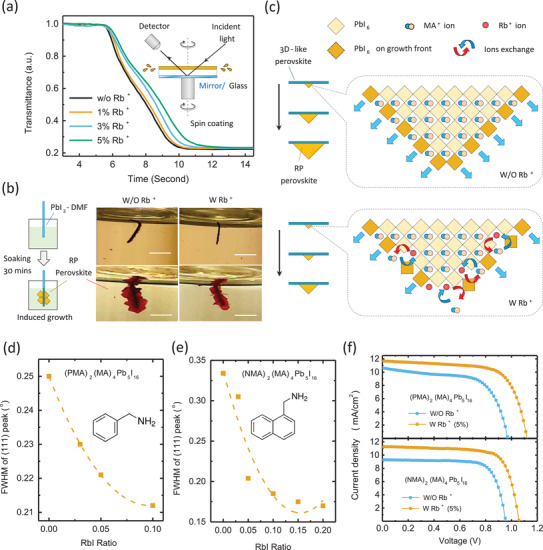
a) Comparison of the transmittance of (PEA)_2_(MA)_4_Pb_5_I_16_films against time during spin coating, in which the decrease in transmittance indicates the formation of perovskite phase. Inset illustrates the in situ transmittance characterization of the (PEA)_2_(MA)_4_Pb_5_I_16_film during spin coating. b) Optical photos of the directional growth of PEA‐based RP crystals on PbI_2_‐DMF solvated phase in the oversaturated precursor solution (<*n*> = 2) without and with Rb^+^ions, where the scale bar is 2 mm. c) Illustration of the retarded crystal growing by Rb^+^ions binding on perovskite surface. d,e) The FWHM of (111) diffraction peaks of PMA‐based and NMA‐based RP perovskite films with different amount of Rb^+^ions added in precursor solution. f) Impacts of Rb^+^ions inhibitor on the*J*–*V*curves of (PMA)_2_(MA)_4_Pb_5_I_16_and (NMA)_2_(MA)_4_Pb_5_I_16_solar cells (under 100 mW cm^−2^AM1.5G illumination), respectively.

Since Rb^+^ ions are not incorporated into perovskite lattice but prefer to attach on the surface of RP perovskite crystals, the retarded crystal growth can be attributed to the surficial blocking effect of Rb^+^ ions. It's known that the ion exchange between alkali metal ions (like Cs^+^, Rb^+^) and organic cations (like MA^+^ and FA^+^) are quite inefficient,^[^ ^38^, ^39^ ^]^ so that the Rb^+^ ions binding with PbI_6_ octahedra would act as barriers to slow down the absorption and diffusion of organic cations (e.g., MA^+^ and PEA^+^) on the crystal surface, which is crucial for crystal growth. An NH_4_Cl‐dissolving experiment was used to support our explanation (Figure S10, Supporting Information). It's known that NH_4_Cl cannot dissolve in pristine DMF solution but can dissolve in PbI_2_:DMF solution because NH_4_Cl can be absorbed on PbI_2_‐DMF colloidal surface and diffuse in to form NH_4_Cl‐PbI_2_‐DMF complexes.^[^ ^40^, ^41^ ^]^ As an interesting contrast, when 5% RbI was added in PbI_2_:DMF solution, the dissolve of NH_4_Cl was significantly retarded (Figure S10, Supporting Information). The slower formation of NH_4_Cl‐PbI_2_‐DMF complexes in the presence of a small amount of Rb^+^ ions proves that the attached Rb^+^ ions on PbI_2_‐DMF colloidal surface slows down the absorption and diffusion of NH_4_^+^ ions into the PbI_2_‐DMF colloidal (see illustration in Figure S10a, Supporting Information). Similarly, Figure 4c schematically compares the growth of RP perovskite without or with Rb^+^ ions, the displacing rate of Rb^+^ ions from inner layer to surface during crystal growth limits the diffusion of PEA^+^ and MA^+^ cations from surface to inner layer, regulating the crystal growth rate.

This Rb^+^ regulated crystal growth is supposed to benefit the crystallinity of PEA‐based RP perovskite with less structural disorders. We tentatively hypothesize that complicating the shape of bulky organic cation would increase the mismatch between crystal growth rate and the assembling rate of bulky organic cations. This is reasonable because the transporting and assembling of organic cations with nonlinear shapes or conjugating segments generally face higher intermolecular friction and hence is slower than that of organic cations with simple shapes (e.g., BA^+^ cation). A slower crystal growth rate regulated by Rb^+^ ions can reduce this mismatch, resulting in RP perovskite crystals with less structural disorders and hence narrowed and enhanced XRD diffraction peaks as shown in Figure 1d–g and Figure S2 (Supporting Information). For a further exploration, we applied this Rb^+^ ions inhibitor to two other RP perovskites based on nonlinear organic cations (i.e., phenylmethylammonium (PMA^+^) and 1‐(2‐naphthyl)‐methanammoniun (NMA^+^)). As shown in Figure 4d,e, gradually increased Rb^+^ ions loading can also reduce the FWHM of the (111) diffraction peaks of PMA‐ and NMA‐based RP perovskites. Figure 4f and Table S1 (Supporting Information) show that using Rb^+^ ions as inhibitor also helps to increase the PCEs of the PMA‐based RP perovskite from 6.5% to 9.0%, and the PCEs of the NMA‐based RP perovskite from 6.7% to 8.8%.

Additional ultraviolet photoelectron spectroscopy (UPS) analysis (Figure S11a, Supporting Information) confirmed that the Rb^+^ ions‐rich region show a Fermi‐level and valance band of −3.94 and −5.59 eV, respectively, which match well with that of the bulk (PEA)_2_(MA)_4_Pb_5_I_16_ (<*n*> = 5) phase (−4.02 and −5.63 eV, respectively). For this reason, the Rb^+^ ions‐rich region could facilitate the hole extraction at the anode/perovskite interface without forming energy barriers for hole transfer (Figure S11b, Supporting Information).

In summary, we prove that using a small amount of crystal growth inhibitor in precursor solution can be a promising strategy to improve the crystallinity of RP type layered perovskites because it can regulate the crystal growth rate, which benefits crystals with higher degree of structural ordering without changing its dominating crystal orientation. To be a superficial crystal growth inhibitor, the additive is supposed to be able to attach at the crystal growing front during the formation process and properly retard the absorption and diffusion of constituent ions. Here we show that Rb^+^ ions with small ionic radius and strong binding effect with PbI_6_ octahedra can play this role in RP perovskites with nonlinear bulky organic cations. For (PEA)_2_(MA)_4_Pb_5_I_16_ based PSCs (<*n*> = 5), adding 5% Rb^+^ ions in precursor solution promotes the PCEs from 12.5% to 14.6%. This increased PCE mainly originated from enhanced carrier recombination lifetimes due to minimized structural disorders in resulted perovskite films. Similar Rb^+^ ion enhanced crystallinity is achieved in other RP perovskites with nonlinear bulky cations (i.e., PMA^+^ and NMA^+^), indicating this method as a general route to regulate the growth of perovskites.

## Experimental Section

##### Materials

Phenylethyl‐ammonium (PEA) and PbI_2_ (99.999%), RbI, bathocuproine (BCP, 99.99%) NH_4_Cl (99.5%) and *N*,*N*‐Dimethylformamide (DMF, 99%) were purchased from Sigma‐Aldrich, Poly(3,4‐ethylenedioxythiophene): poly(styrenesulfonate) (PEDOT:PSS) aqueous solution was purchased from Heraeus Ltd,[6,6]‐Phenyl‐C61‐butyric acid methyl ester (PC_61_BM) was purchase from Lumtec. Methylammonium iodide (MAI) were purchased from Greatcell Solar Ltd. RP perovskite precursor solutions (1.0 m) with various organic spacer cations were prepared by PEA, MAI, PbI_2_, and NH_4_Cl in a molar ratio of 2:4:5:1:1 in DMF.

##### Characterization

All the *J*–*V* curves of device were measured by using a Keithley 2400 with a scan rate of 0.2 V s^−1^, and scan from −0.2 to 1.2 V under illumination by a 100 mW cm^−2^ AM1.5G illumination from an AAA class solar simulator (Enli Technology Co., Ltd.). Silicon cells what was certificated by NREL are used for calibration. Absorption spectra were measured using a UV–visible Spectrometer (Thermo Evolution 201) with the spectral range of 300–1100 nm. X‐ray diffraction (XRD) measurements were carried out with a Siemens D500 Bruke X‐ray diffractometer (Cu K*α* radiation, *λ* = 1.5406 Å). Scanning electron microscopy (SEM) characterizations were performed by using a scanning electron microscope (TESCAN MIRA3 LMU) at an acceleration voltage of 20 keV. X‐Ray energy dispersive spectroscopy (EDS) analysis was obtained by an X‐Ray energy dispersive spectrometer (TESCAN MIRA3 LMU) with electron beam acceleration at 20 kV. GI‐XRD measurement was performed on a Xenocs Xeuss 2.0 system. The wavelength of the X‐ray beam is 0.154 nm with a flux of ≈4.6 × 10^7^ photons s^−1^ and an illumination area of 1.2 × 1.2 mm^2^. The incident angle of the X‐ray beam was set as 0.5°. The 2D‐ GI‐XRD patterns were collected by a Pilatus 300K detector and analyzed using the software package FIT2D. For X‐ray photoelectron spectroscopy (XPS) measurement, the X‐ray source was operated at 100 W with 40 eV pass energy and 100 meV scanning step unless otherwise specified. The angle between the incident photon and the emitted photoelectron direction was 45° for UPS and XPS. The binding energies (EB) of all spectra were calibrated to the Fermi level (EF) of the energy analyzer. Electrochemical impedance spectra (EIS) of the devices were characterized using an electrochemical workstation with the measured frequency range from 2 MHz to 20 Hz in dark conditions.

##### Device Fabrication, Measurements, and Simulation

ITO glass (sheet resistance ≤15 Ω cm^−2^) was cleaned consecutively with deionized water, acetone and isopropanol ultrasonic bath for 15 min, respectively, blown dry with nitrogen, and treated by UV–ozone for 10 min before use. PEDOT:PSS was spin‐coated onto the prepared ITO at 3000 rpm for 30 s, it was then annealed in air at 125 °C for 30 min. The 50 µL RP perovskite precursor was spin‐coated onto PEDOT:PSS substrate at 5000 rpm for 40 s, then heated at 100 °C for 60 min. Thereafter, PCBM (15 mg mL^−1^ in chlorobenzene) was spin‐coated onto RP perovskite film at 3000 rpm for 30 s. BCP was deposited on the substrate by vacuum evaporation at about 7 nm. Finally, Cu electrode with a thickness of about 70 nm was deposited in the vacuum evaporation chamber.

## Conflict of Interest

The authors declare no conflict of interest.

## Supporting information

Supporting Information
