# Supplementary material for: Rubidium Ions Enhanced Crystallinity for Ruddlesden–Popper Perovskites
Source: Adv Sci (Weinh). 2020 Nov 13;7(24):2002445. doi: 10.1002/advs.202002445 (PMC7740094; doi:10.1002/advs.202002445)
Supplement: Supplementary file 1 — Supporting Information [file ADVS-7-2002445-s001.pdf]

© 2020 Wiley-VCH GmbH

ADVANCED SCIENCE
Open Access

Supporting Information

for *Adv. Sci.,* DOI: 10.1002/advs.202002445

Rubidium Ions Enhanced Crystallinity for

Ruddlesden-Popper Perovskites

Shaowen Cui, Jifei Wang, Haipeng Xie, Yuan Zhao,
Zhimin Li, Shiqiang Luo, Lili Ke, Yongli Gao,
Ke Meng, Liming Ding,* and Yongbo Yuan*

Supporting Information

Rubidium Ions Enhanced Crystallinity for Ruddlesden–Popper Perovskites

Shaowen Cui[#], Jifei Wang[#], Haipeng Xie, Yuan Zhao, Zhimin Li, Shiqiang Luo, Lili Ke, Yongli Gao, Ke Meng, Liming Ding* and Yongbo Yuan**

S. Cui, Dr. J. Wang. Dr. H. Xie, Dr. Y. Zhao, Dr. S. Luo, Dr. L. Ke, Prof. Y. Gao, Prof. Y. Yuan
Hunan Key Laboratory of Super Microstructure and Ultrafast Process, School of Physics and Electronics, Central South University, Changsha, Hunan 410083, China.
E-mail: yuanyb@csu.edu.cn.

Prof. Y. Yuan
State Key Laboratory of Powder Metallurgy, Central South University, Changsha, Hunan 410083, P. R. China.

Dr. Z. Li, Prof. K. Meng
School of Physical Science and Technology, Shanghai Tech University, Shanghai 201210, China
E-mail: mengke@shanghaitech.edu.cn

Prof. L. Ding
Center for Excellence in Nanoscience (CAS), Key Laboratory of Nanosystem and Hierarchical Fabrication (CAS), National Center for Nanoscience and Technology, Beijing 100190, China.
E-mail: ding@nanoctr.cn

[#] These authors contributed equally to this work

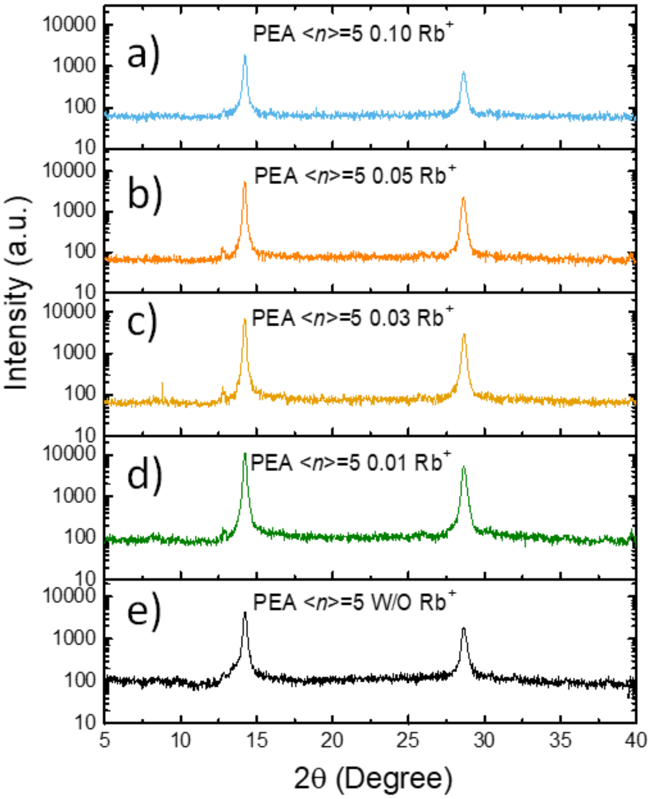

Figure S1. a-e) The X-ray diffraction (XRD) characterization of the $(PEA)_2(MA)_4Pb_5I_{16}$ (<*n*>= 5) films with 0.10 (a), 0.05 (b), 0.03 (c), 0.01 (d) Rb^+ ions and without Rb^+ ions (e).

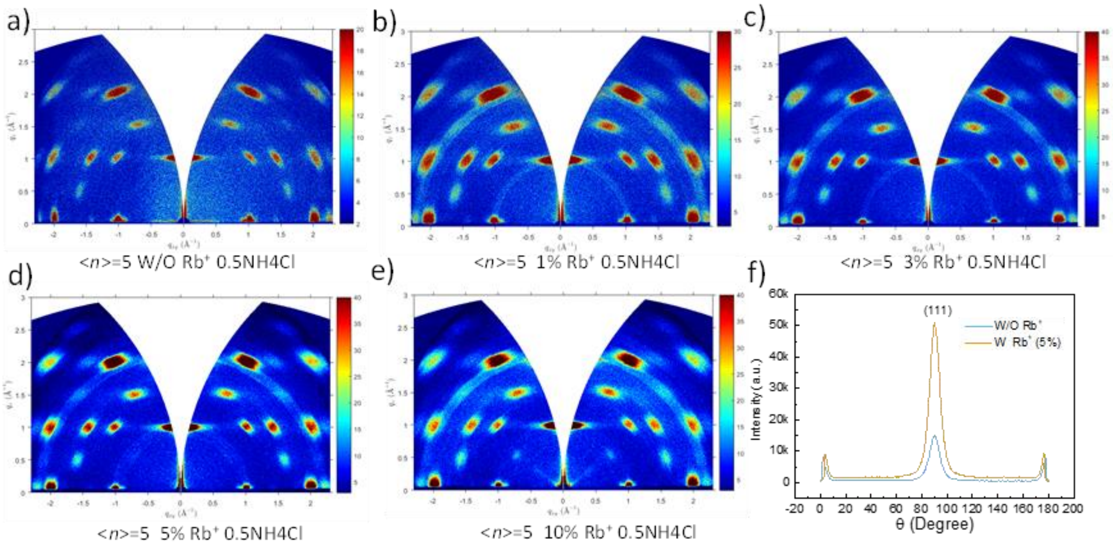

Figure S2. a-f) The GIWAXS patterns of the $(PEA)_2(MA)_4Pb_5I_{16}$ perovskite films without adding Rb^+ ions (a), and with 1% (b), 3% (c), 5% (d), 10% (e) Rb^+ ions. (f) The (111) diffraction peaks intensity of PEA-based RP perovskite without and with 5% Rb^+ ions.

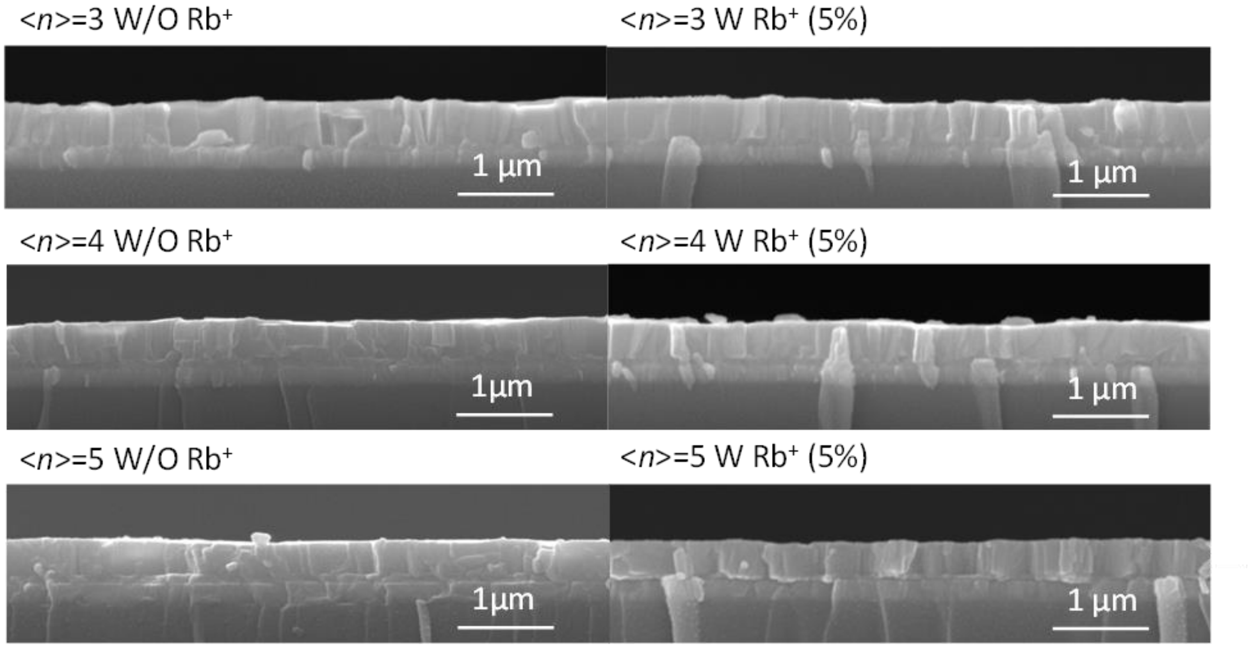

Figure S3. Cross-sectional SEM images of PEA-based RP perovskite films (<*n*>=3, 4, and 5) without and with 5% Rb^+.

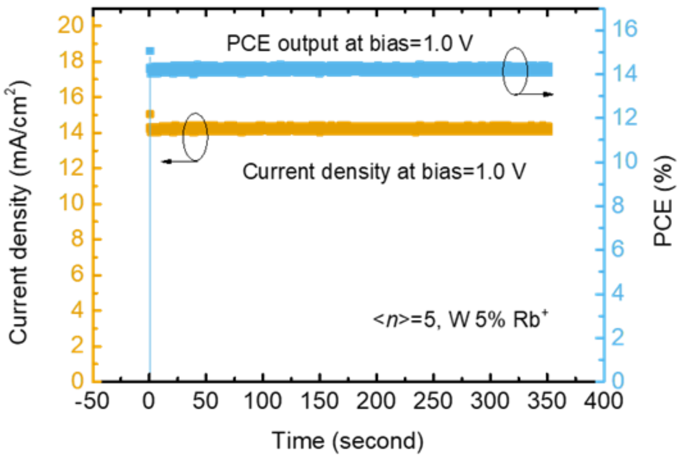

Figure S4. The stabilized power output of the champion device (<*n*>=5, 5% Rb^+) at a fixed maximum power point (MPP) voltage as a function of time.

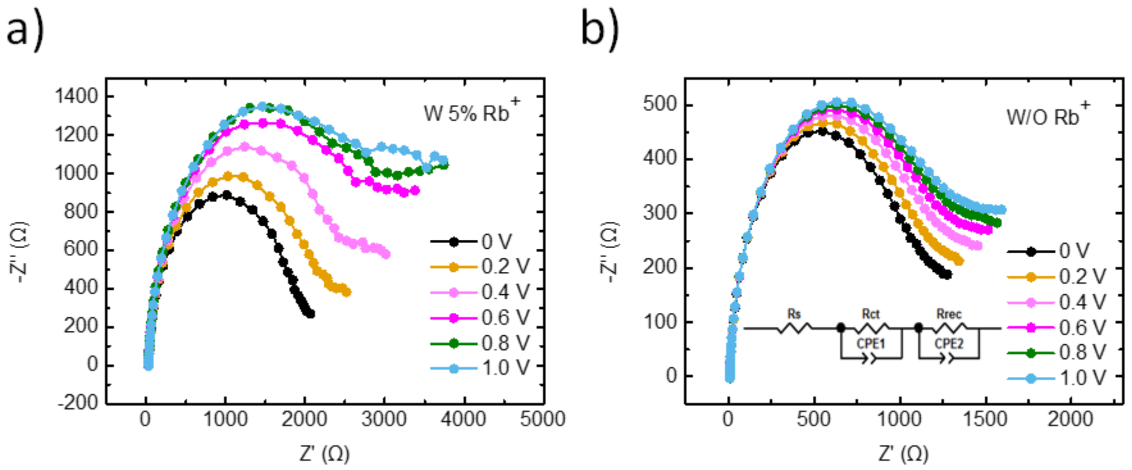

Figure S5. a,b) Nyquist plots of impedance spectra for PSCs (<*n*>=5) with 5% Rb^+ ions (a) and without Rb^+ ions (b) at different voltages. Inset: The equivalent circuit model for the solar cells.

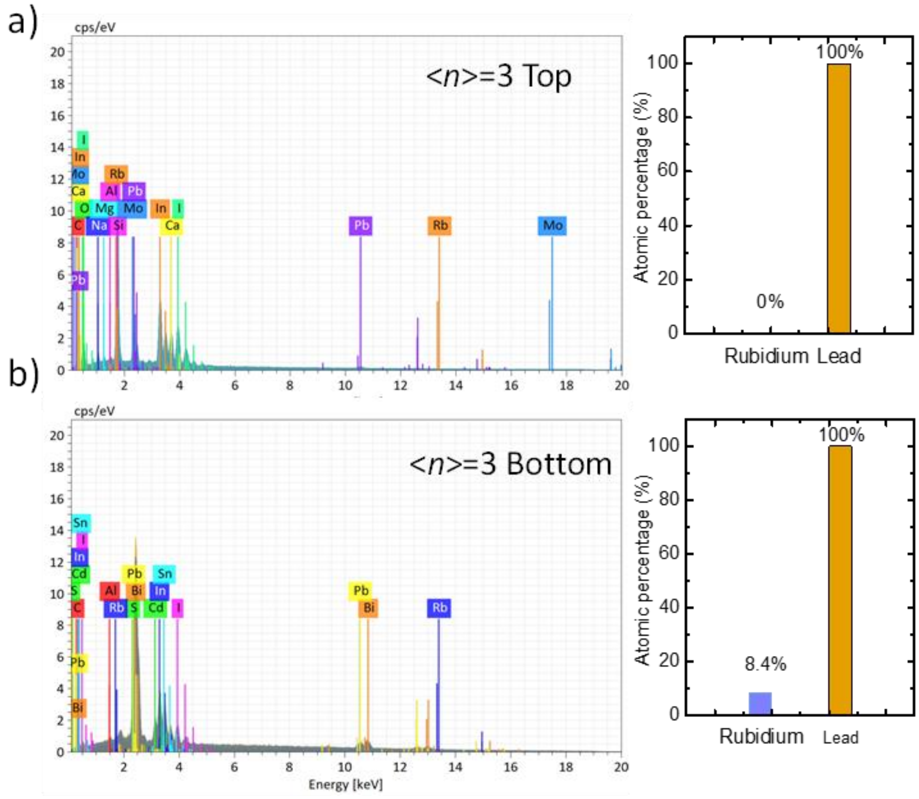

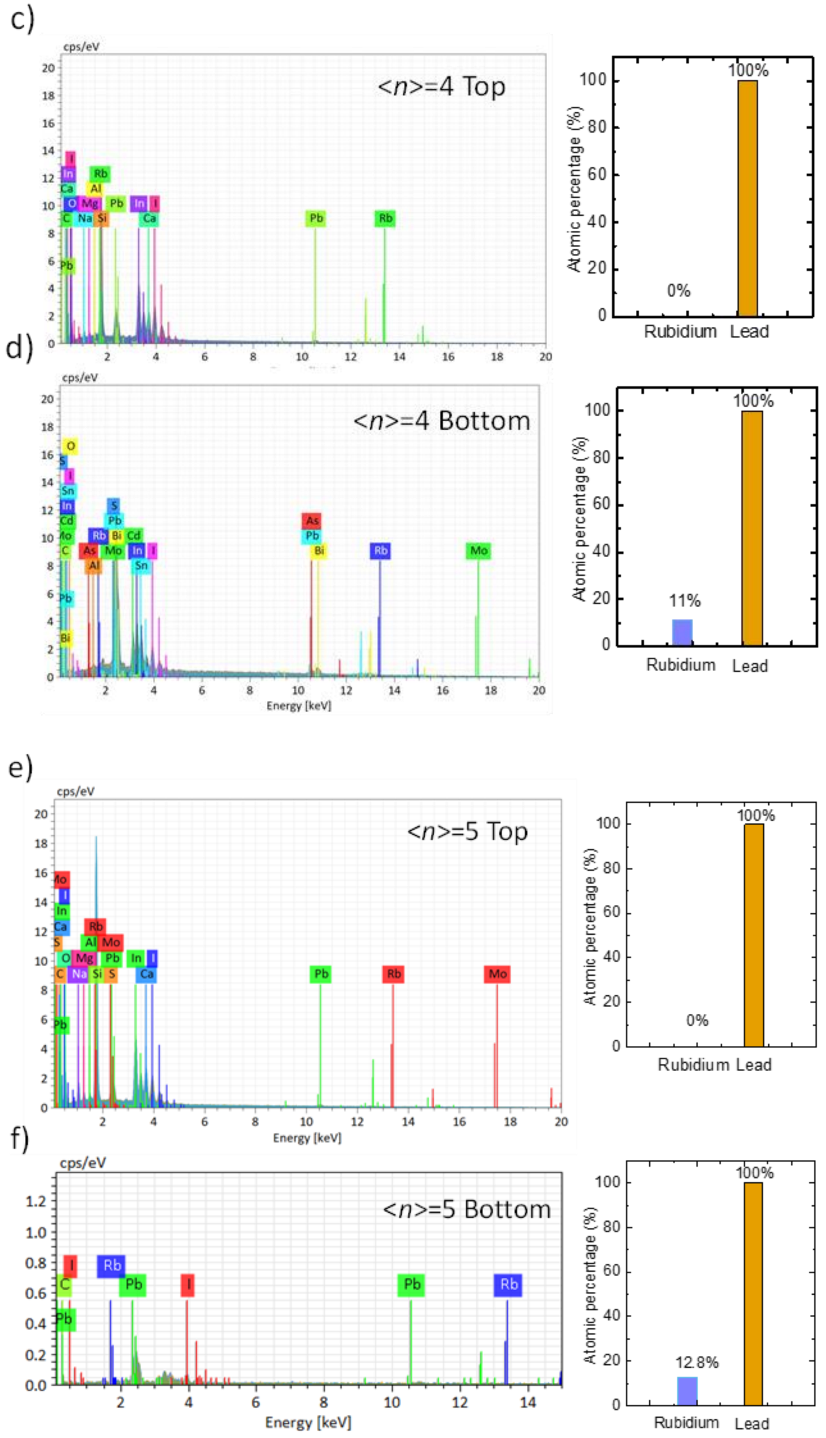

Figure S6. a-f) X-Ray Energy Dispersive Spectroscopy (EDS) of the top and bottom of $(PEA)_2(MA)_{n-1}Pb_nI_{3n+1}$ films (<*n*>=3, 4, and 5). The atomic percentage ratio of Rb to Pb was illustrated on the right of the corresponding EDS testing figure.

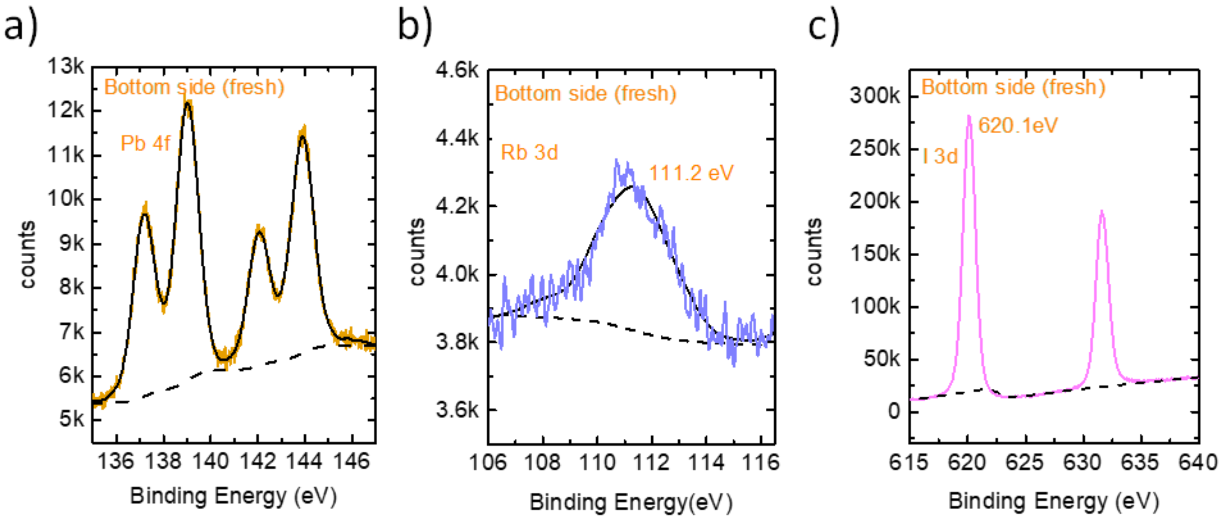

Figure S7. High-resolution XPS spectra of Pb 4f (a), Rb 3d (b) and I $3d_{5/2}$ (c) for bottom side of the $(PEA)_2(MA)_4Pb_5I_{16}$ films, the ratio of the integral area of Rb 3d to Pb 4f is 221:3081. Given the atomic sensitivity factors, the molar ratio of Rb to Pb is 38:100. The binding Energy of I $3d_{5/2}$ and Rb 3d is 620.1 eV and 111.2 eV, which is slightly higher than I $3d_{5/2}$ (619.5 eV) and Rb 3d (110.4 eV) of RbI, respectively.

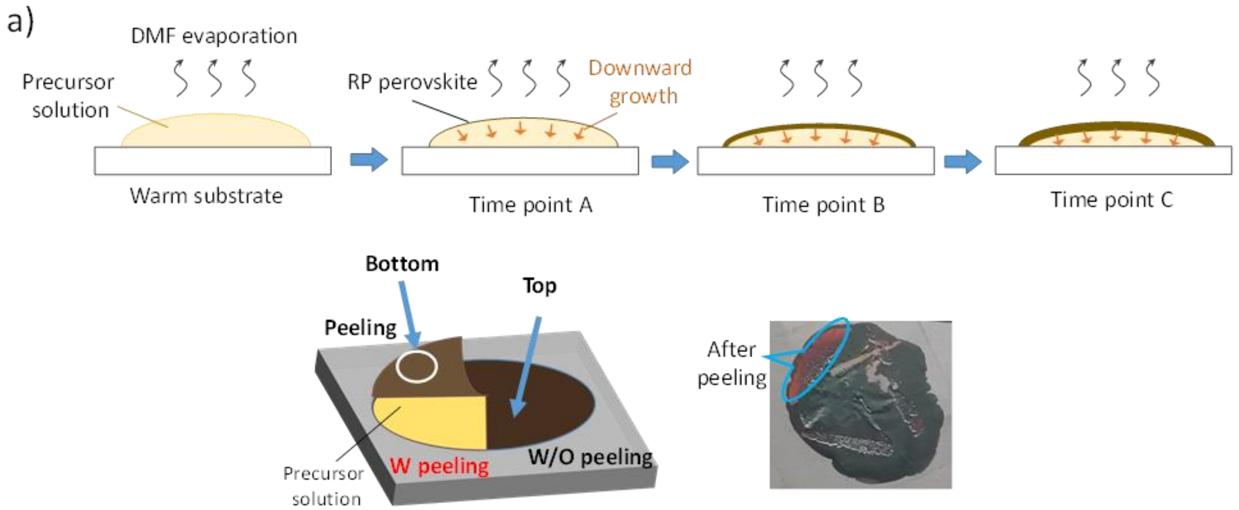

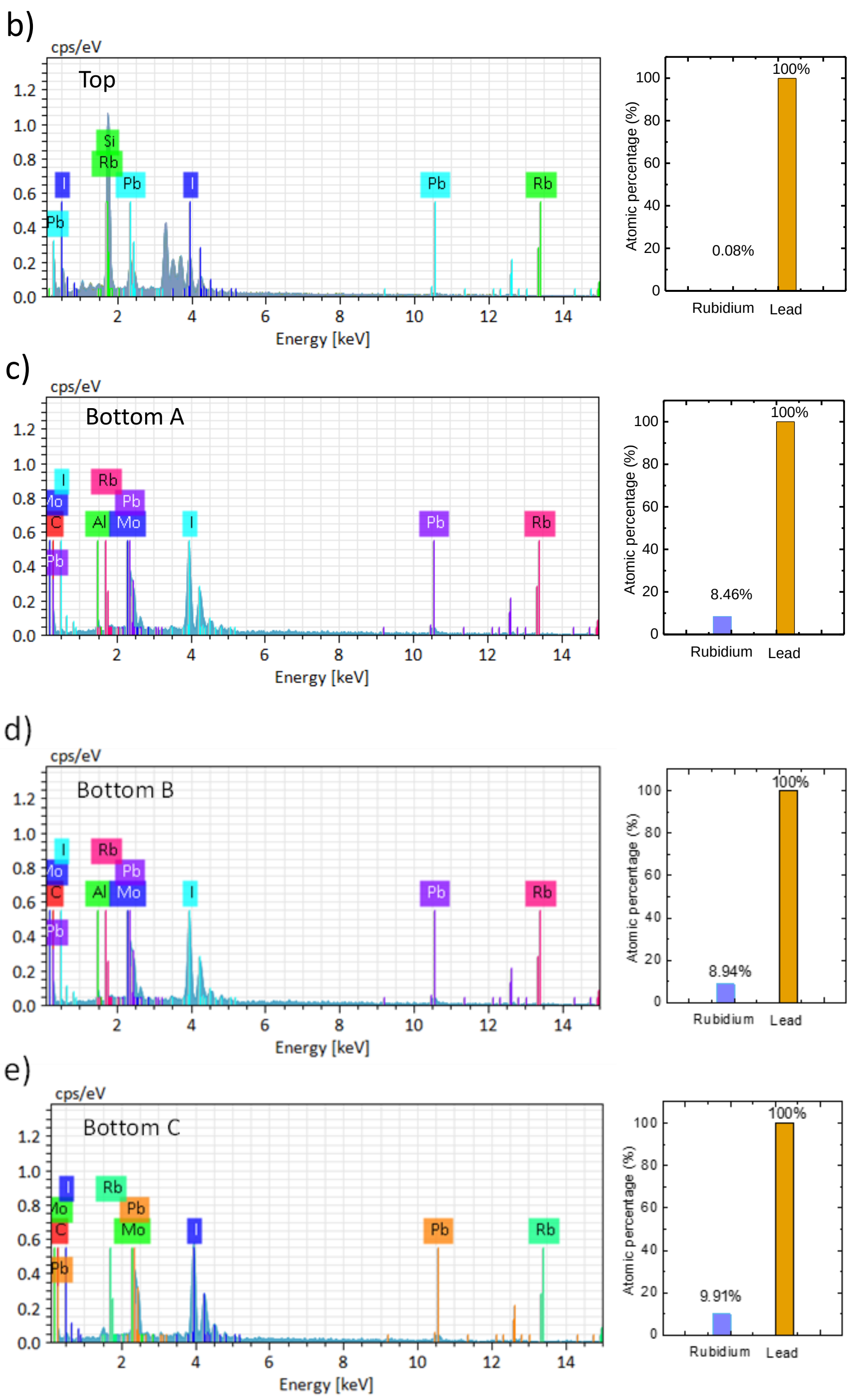

Figure S8. a) Illustration of the top-crust peeling-off test, in which the RP perovskite films formed on liquid/air interface were peeled off for EDS testing. The precursor solution of PEA-based RP perovskites was dropped on a heating substrate, after which the RP perovskite film preferred to form at the liquid/air interface and adopted downward growth; b) EDS spectrum of the top of the RP perovskite film after heating for 30 mins; c-e) EDS spectra of the bottom of the peeled RP perovskite film after heating for 5 mins (c), 10 mins (d) and 30 mins (e). All films were formed on top of the solution and heated at 80 ℃. The atomic percentage ratio of Rb to Pb was illustrated on the right of the corresponding EDS testing figure.

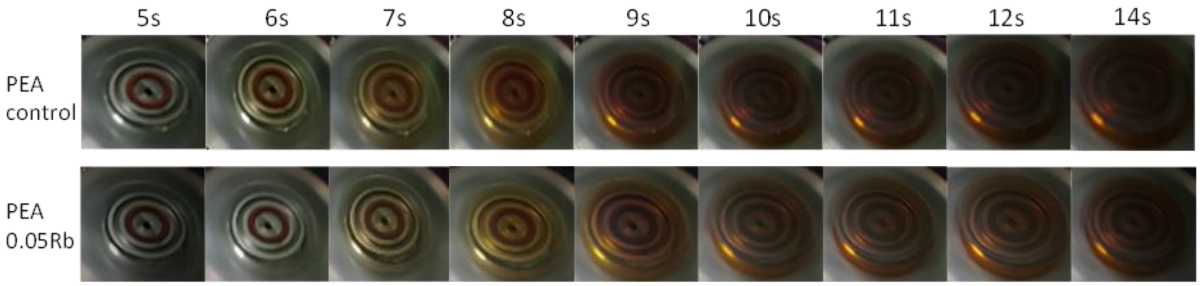

Figure S9. The diagram of color change process of the $(PEA)_2(MA)_4Pb_5I_{16}$ films without and with 5% Rb^+ in the process of spin coating over time. At 6s, 7s, 8s and 9s, the color change of the film with Rb^+ was slower than that of the film without Rb^+.

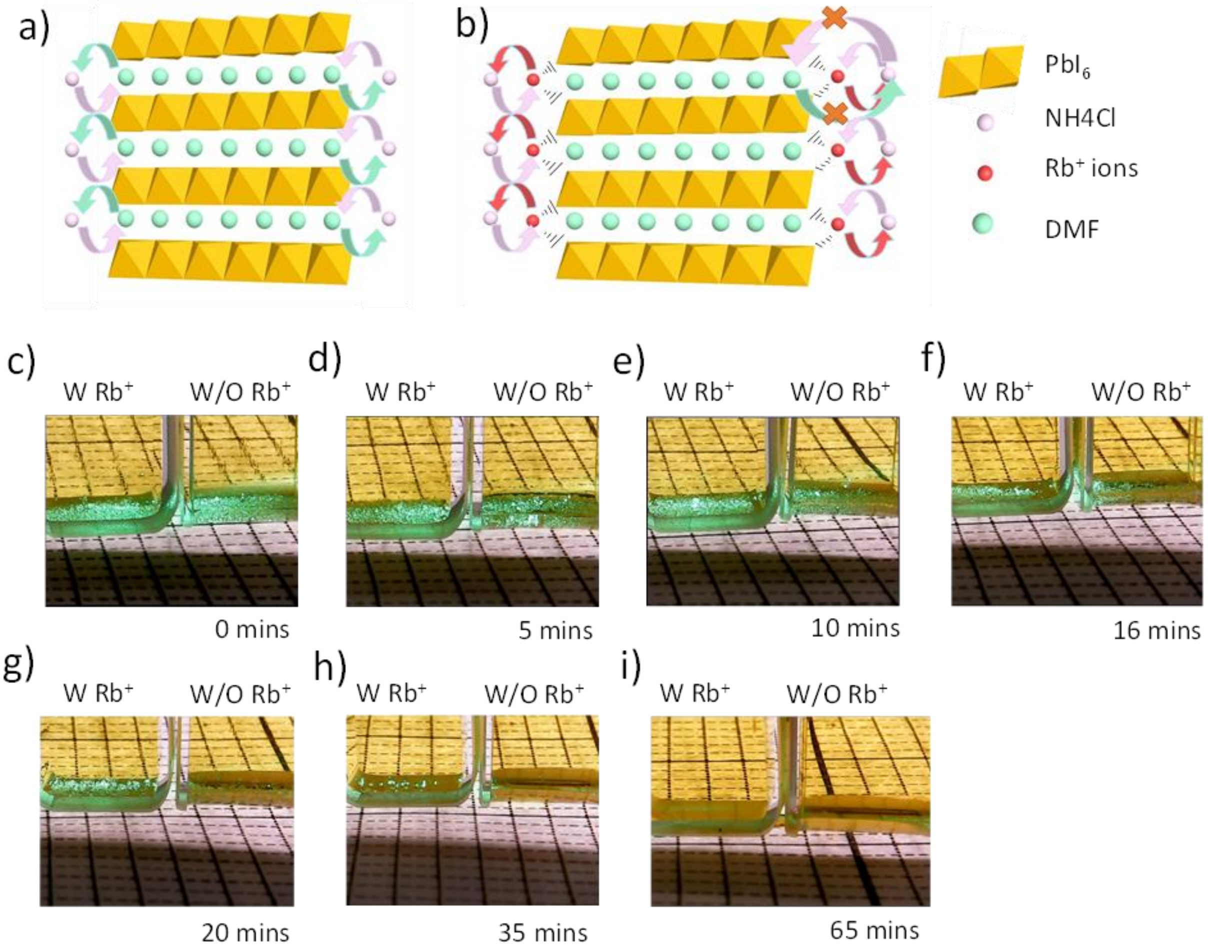

Figure S10. a,b) The Schematic diagram of the dissolution process of NH_4Cl in PbI_2:DMF solution (a) without and (b) with 5% RbI. c-i) Comparison of the dissolution rate of NH_4Cl in PbI_2:DMF solution with and without 5% RbI after heating for 0 min (c), 5 mins (d), 10 mins (e), 15 mins (f), 20 mins (g), 35 mins (h) and 65 mins (i). The powder (illuminated by green light) at the bottom of vials in Figure S10c-10i are NH_4Cl.

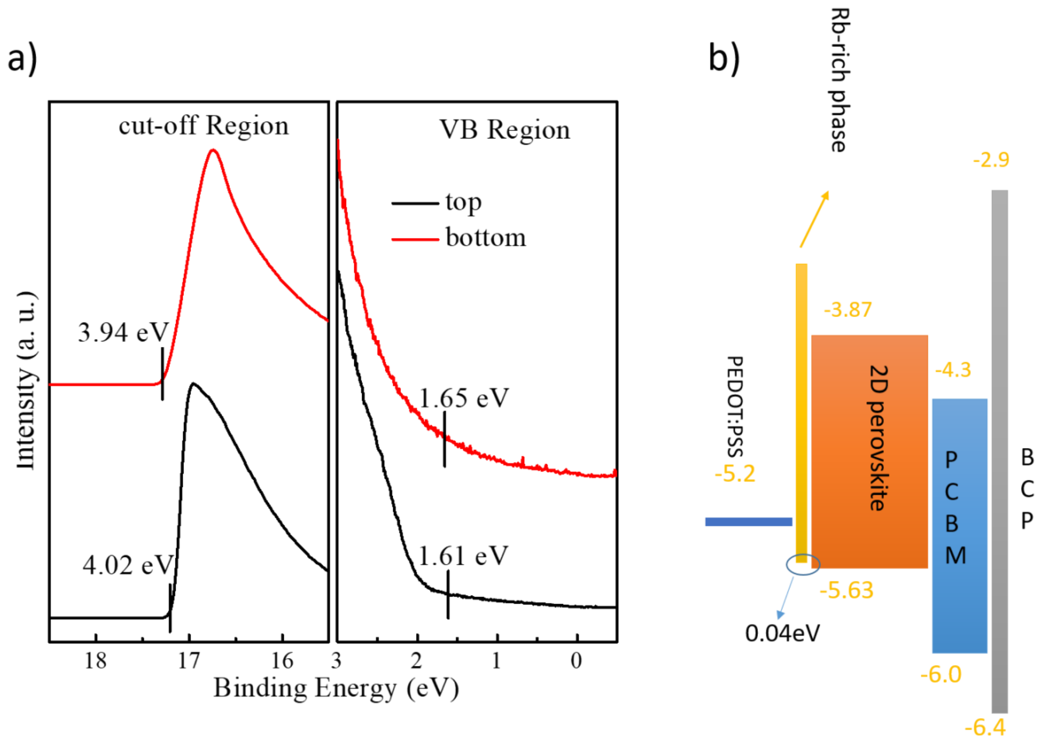

Figure S11. a) Ultraviolet photoelectron spectroscopy (UPS) of the top and bottom regions of the PEA-based 2D perovskite, where the top regions is dominated by the $(PEA)_2(MA)_4Pb_5I_{16}$ phase without Rb^+ ions and the bottom region is dominated by Rb^+ ions-rich phase. The locations of Fermi-level determined from the cut-off binding energy of secondary electron in UPS measurements; the locations of valence band relative to the Fermi-level identified by the onset of the UPS spectra; b) Illustration of the energy diagram of PEA-based RP perovskite solar cells with Rb^+ ion-rich region located at the anode/PVK interface.

Table S1. Photovoltaic parameters of the $(PEA)_2(MA)_4Pb_5I_{16}$, $(PMA)_2(MA)_4Pb_5I_{16}$, $(NMA)_2(MA)_4Pb_5I_{16}$ perovskite solar cells without and with 5 % Rb^+. The device structure is ITO /PEDOT:PSS / RP perovskites /$PC_{61}BM$ /BCP /Cu.

RP perovskites	V_{oc} (V)	J_{sc} (mA/cm^2)	FF	PCE (%)
W/O Rb^+ $(PEA)_2(MA)_4Pb_5I_{16}$	1.13	16.10	68.5	12.5
W 5% Rb^+ $(PEA)_2(MA)_4Pb_5I_{16}$	1.22	15.77	75.8	14.6
W/O Rb^+ $(PEA)_2(MA)_3Pb_4I_{13}$	1.12	15.30	65.1	11.3
W 5% Rb^+ $(PEA)_2(MA)_3Pb_4I_{13}$	1.17	14.90	71.2	12.3
W/O Rb^+ $(PEA)_2(MA)_2Pb_3I_{10}$	1.20	10.78	57.8	7.50
W 5% Rb^+ $(PEA)_2(MA)_2Pb_3I_{10}$	1.20	13.72	57.6	9.5
W/O Rb^+ $(PMA)_2(MA)_4Pb_5I_{16}$	0.97	10.63	63.3	6.5
W 5% Rb^+ $(PMA)_2(MA)_4Pb_5I_{16}$	1.12	11.70	68.7	9.0
W/O Rb^+ $(NMA)_2(MA)_4Pb_5I_{16}$	0.95	9.30	75.2	6.7
W 5% Rb^+ $(NMA)_2(MA)_4Pb_5I_{16}$	1.06	11.32	73.6	8.8

Table S2. Summary of the Rb/Pb atomic ratio of the top and bottom of the $(PEA)_2(MA)_{n-1}Pb_nI_{3n+1}$ films ($\langle n \rangle$ =3, 4, 5) determined from EDS analysis.

Atomic ratio (Rb/Pb)	$\langle n \rangle$=3	$\langle n \rangle$=4	$\langle n \rangle$=5
Top	0	0	0
Bottom	8.4:100	11:100	12.8:100

Table S3. Summary of the Rb/Pb atomic ratio of the top of the RP perovskite films after heating for 30 mins and the Rb/Pb atomic ratio of the bottom of RP perovskite films after heating for 5 mins, 10 mins and 30 mins.

Samples	30 mins (Top)	5 mins (Bottom)	10 mins (Bottom)	30 mins (Bottom)
Atomic ratio (Rb/Pb)	0.80:100	8.46:100	8.94:100	9.91:100

Supplemental Note 1.

In-situ transmittance characterization of the perovskites films, we use the calibrated silicon diodes as the reflected light signal detector, a monochromatic light with wavelength of 520 nm as incident light; Aluminium (100 nm) was deposited onto the back of the PEDOT:PSS-coated ITO substrate as a highly reflective film, then the 50 μL perovskite precursor was spin coated onto the PEDOT:PSS substrate at 5000 rpm for 40 s. The whole process takes place in the nitrogen glove box.
